# High Latent TB Infection Rate and Associated Risk Factors in the Eastern China of Low TB Incidence

**DOI:** 10.1371/journal.pone.0141511

**Published:** 2015-10-27

**Authors:** Cheng Chen, Tao Zhu, Zhijian Wang, Hong Peng, Wen Kong, Yang Zhou, Yan Shao, Limei Zhu, Wei Lu

**Affiliations:** 1 Department of Chronic Infectious Disease, Center for Disease Control and Prevention of Jiangsu Province, Nanjing, China; 2 Department of tuberculosis control, Center for Disease Control and Prevention of Danyang County, Danyang, China; University of Minnesota, UNITED STATES

## Abstract

**Objectives:**

To disclose the associated risk factors for latent tuberculosis infection (LTBI) and the current situation of LTBI in the eastern China.

**Methods:**

A cross-sectional study was undertaken to evaluate the LTBI rate and risk factors.

**Results:**

A total of 5305 subjects were finally included, with the IGRA positive rate of 19.98% (1060/5305). The LTBI rates were increasing with age (ORs were in significance from 6.60 to 20.92). Male gender significantly increased the risk of LTBI by 0.52 fold (OR = 1.52). Both smoking and drinking significantly increased the risk of LTBI (OR = 1.83 and OR = 1.67, respectively). Meanwhile, overweight and close contact with tuberculosis were risk factors for LTBI (OR = 1.36 and OR = 2.38, respectively). However, higher level of education and BCG vaccination lowered the risk of LTBI (OR = 0.16 and OR = 0.39, respectively). The multivariate logistic regression showed that age, male gender, smoking, overweight and close contacting with tuberculosis were risk factors for LTBI, but BCG vaccination was a protective factor for LTBI.

**Conclusions:**

BCG vaccination exerted protective effect on tuberculosis. However, LTBI rate in the Chinese rural area was critical and subjects above 30 years, male, smoking, overweight and close contact with tuberculosis wound be the targets for LTBI screening and source of tuberculosis.

## Introduction

Latent tuberculosis infection (LTBI) is defined as a state of persistent mycobacteria-specific T-cell responses without clinical evidence of tuberculosis [1]. It was estimated that one-third of the world population was infected by *M*. tuberculosis [2], and most of the infected people had no signs and symptoms of tuberculosis. Although LTBI people were not transmitters, some of the infected people were at a risk of developing active disease of tuberculosis during their lifetime, and it is reported that 5–10% of the LTBI people would finally turn out to be of tuberculosis [3]. China has the second largest number of tuberculosis patients in the world [4]. To make things worse, the LTBI population would potentially contribute to the aggressively increased new tuberculosis incidences and which may be greatly counted on the LTBI rate of the population, especially for the rural areas of China because tuberculosis patients from rural areas accounted for a major proportion of tuberculosis incidences. Thus, to accurately elucidate the current LTBI in rural areas of China is of critically important for tuberculosis control. Jiangsu Province was located in the eastern part of China with relatively lower incidence of tuberculosis [5], and it is worthwhile to reveal the situation of LTBI in this area.

Currently, LTBI screening was recommended on those target populations at high risk of developing tuberculosis, such as patients receiving tumor necrosis factor treatment, co-infection with HIV and children aged less than 5 years [6–8]. Meanwhile, prophylaxis treatment method was an alternative for tuberculosis control for the high risk populations [9, 10]. However, the extensive testing LTBI for the general population was not affordable, especially in the health resource limited regions. In our study, we also intend to find out the related risk factors for LTBI in this study, and provide evidences for adopting necessary interventions on the general population to decrease the LTBI rate.

## Methods

### Study design and participants

This cross-sectional study was undertaken from 1 July to 31 July in 2013 of Danyang County, Jiangsu Province. Two villages of Danyang County were chosen to conduct the study. Meanwhile, two steps of population screening were carried out in our study. Firstly, we interviewed the residence population of the two villages with a face to face way. The residence population of the study site was 7311. Among the residence population, 110 children with age less than 5 years and 4 pregnant women were excluded. There is no present active pulmonary tuberculosis in the residence population. For the first step survey, 1199 of the qualified subjects declined or could not complete in the study period. Thus, the eligible population included in the baseline survey was 5998. For the next step, we conducted investigation on the eligible subjects and the IGRA test for each subject. During the investigation, 520 eligible subjects did not join the investigation. The following 116 subjects who finished the investigation were also excluded from this cross-sectional study (among the 116 subject, 12 subjects presented clinically suspected pulmonary tuberculosis signs and symptoms and self-reported a history of tuberculosis simultaneously): one subject with absent result of IGRA, 16 subjects with absent results of digital chest radiography, 45 subjects reported a history of tuberculosis, 66 subjects presented clinically suspected pulmonary tuberculosis. Finally, 5361 subjects were selected for evaluation of the prevalence of LTBI, with a response rate of 73.33% (5361/7311). The study was approved by the ethics committee of the Institute of Pathogen Biology, Chinese Academy of Medical Sciences, and all the participants provided written informed consent before undergoing the investigation (For those enrolled minors or children above 5 years old, their parents or guardians provided written informed consent on behalf of them before the investigation). Smoking was defined as tobacco consumption more than 5 cigarettes per month and lasted for at least 6 months consecutively. Drinking was defined as alcohol consumption more than 100 mL per month in the last year. Close contact with tuberculosis was considered as contacting tuberculosis patients in household or workplace. The body-mass index (BMI) was classified as underweight (BMI < 18.5 Kg/m^2^), normal weight (BMI ≥ 18.5 Kg/m^2^ and BMI < 23 Kg/m^2^), overweight (BMI ≥ 23 Kg/m^2^ and BMI < 27.5 Kg/m^2^) and obesity (BMI ≥ 27.5 Kg/m^2^) [11].

### Determination of LTBI

Each subject provided approximate 3 mL venous blood sample, and the interferon-gamma release assay (QuantiFERON-TB Gold In-Tube [QFT; Qiagen, Valencia, CA, USA]) was used to evaluate the status of LTBI. The procedure of conducting QFT follows the instructions provided by Qiagen and the methodology of QFT can be referred to the review of Whitworth et al. [12]. 5% of the incubation samples were randomly selected to repeat for consistency, and all the results were 100% consistent with the primary results.

### Exclusion of pulmonary tuberculosis patients and clinically suspected pulmonary tuberculosis

Pulmonary tuberculosis was diagnosed by positive culture and X-ray manifestation of tuberculosis. Those subjects with abnormal digital chest radiography which indicated active pulmonary tuberculosis but without bacteriological evidence were considered as clinically suspected pulmonary tuberculosis patients. In this study, all the pulmonary tuberculosis cases and clinically suspected pulmonary tuberculosis subjects were excluded for the LTBI rate evaluation.

### Statistical analysis

The between-group demographics were compared by person χ^2^ test or fisher exact test for categorical data. The association of risk factors among QTF positive group and QFT negative group was estimated by computing the odds ratio (OR) and 95% confidence intervals (CI) from both univariate and multivariate logistic regression analyses, and the dummy variable was used for those variables with more than two stratifications. *P* value less than 0.05 was considered as statistically significant. All the analysis were performed by Stata software (Version 13.0, StataCorp, Texas, USA)

## Results

**Fig 1:**
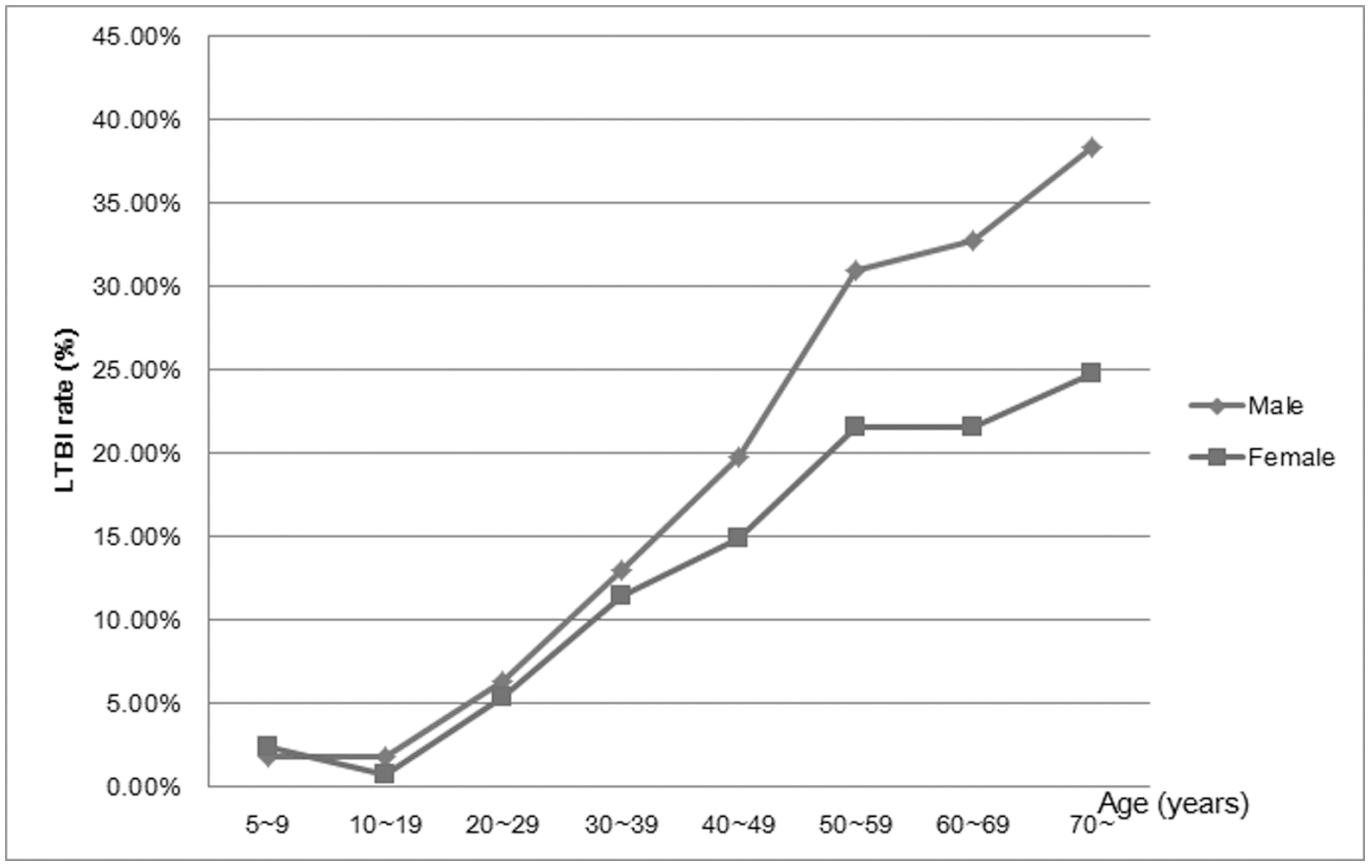
the LTBI rate between male and female subjects along with age groups. Abbreviations: LTBI, latent tuberculosis infection

Of the 5361 participant with valid investigation and receiving QTF test, 56 of them came out of indeterminate QFT result. Thus, 5305 subjects were finally analyzed for evaluating the LTBI rate and associated risk factors (S1 Dataset). The positive LTBI rate determined by QFT in our study was 19.98% (1060/5305). As shown in Table 1, all the participants were above 5 years old, so we classified the age in to six groups by every ten years from 10 to 70, and finally the age was categorized into eight groups. When taking the 5–9 years class as the reference group, we found that since 30 years age groups, the risk of LTBI significant increased with ORs from 6.60 to 20.92. In this cross-sectional study, the male subjects constituted 46.5% of the total population. However, we found the proportion of male subjects in LTBI positive subjects was more (54.9%) than those in LTBI negative groups (44.4%), the OR showed that male subjects increased the risk of LTBI by 0.52 fold (OR = 1.52, 95%CI, 1.33–1.75). Meanwhile, as shown in Table 2 and Fig 1, the LTBI rate was increasing among male and female subjects along with age growing, and the LTBI rate among male subjects was significantly increased than female subjects from 40–49 years category. The education distribution showed that the higher education level lowered the risk of LTBI when compared to the primary or lower levels (OR = 0.16, 95%CI, 0.08–0.32 for college or higher levels). Smoking significantly increased the risk of LTBI by 0.83 fold (OR = 1.83, 95%CI, 1.59–2.10), while alcohol drinking also significantly increased the risk of LTBI by 0.67 fold (OR = 1.67, 95%CI, 1.43–1.94). Taking the normal weight as the reference, the BMI below 18.5 Kg/m^2^ significantly decreased the risk of LTBI by 0.35 fold (OR = 0.65, 95%CI, 0.46–0.92), while the overweight significantly increased the risk of LTBI by 0.36 fold (OR = 1.36, 95%CI, 1.17–1.57). However, the obesity was not in association with LTBI (*P* = 0.1055). The self-reported close contact with tuberculosis patients was in significant association with LTBI (OR = 2.38, 95%CI, 1.20–4.75), and subjects with bacillus Calmette-Guérin (BCG) scar were at a lower risk of LTBI (OR = 0.39, 95%CI, 0.34–0.46). The family history of tuberculosis was not in association with LTBI in our study (*P* = 0.1126). In multivariate logistic regression analysis (Table 3), we put the following variables concerning LTBI in the model, and only variables with significant result were finally selected into the model: age (every 10 years), gender (Female = 0, Male = 1), BMI (BMI ≥18.5 Kg/m^2^ and BMI <23 Kg/m^2^ (reference) = 0, BMI<18.5 Kg/m^2^ = 1, BMI ≥23 Kg/m^2^ and BMI<27.5 Kg/m^2^ = 2, BMI ≥27.5 Kg/m^2^ = 3), smoking (No = 0, Yes = 1), drinking (No = 0, Yes = 1), Family history of tuberculosis (No = 0, Yes = 1), close contact with tuberculosis (No = 0, Yes = 1) and BCG scar (No = 0, Yes = 1). As a result, we found the age with every 10 years increase (OR = 1.03, 95%CI, 1.03–1.04), male (OR = 1.32, 95%CI, 1.08–1.43), BMI between 23 and 27.5 Kg/m^2^ (OR = 1.24, 95%CI = 1.08–1.43), smoking (OR = 1.41, 95%CI, 1.16–1.71) and close contract with tuberculosis (OR = 2.10, 95%CI, 1.03–4.30) were risk factors for LTBI, while only BCG scar was a protective factor for LTBI (OR = 0.79, 95%CI, 0.65–0.97).

**Table 1:** Baseline characteristics of the enrolled population and the comparisons between QFT positive and QFT negative populations.

Characteristics	Total		QFT Negative		QFT Positive		OR	P Value
Age (years)	n	%	n	%	n	%		
5–9	98	1.8	96	2.3	2	0.2	reference	
10–19	295	5.6	291	6.9	4	0.4	0.66(0.12–3.66)	0.6346
20–29	397	7.5	374	8.8	23	2.2	2.95(0.68–12.74)	0.1468
30–39	463	8.7	407	9.6	56	5.3	6.60(1.58–27.54)	0.0096
40–49	1293	24.4	1073	25.3	220	20.8	9.84(2.41–40.22)	0.0015
50–59	1136	21.4	840	19.8	296	27.9	16.91(4.14–69.04)	<0.0001
60–69	1030	19.4	751	17.7	279	26.3	17.83(4.37–72.82)	<0.0001
≥70	593	11.2	413	9.7	180	17.0	20.92(5.10–85.78)	<0.0001
Gender								
Female	2838	53.5	2360	55.6	478	45.1	reference	<0.0001
Male	2467	46.5	1885	44.4	582	54.9	1.52(1.33–1.75)	
Education								
Primary school or lower	2356	44.4	1799	42.4	557	52.5	reference	
Middle school	2090	39.4	1709	40.3	381	35.9	0.72(0.62–0.83)	<0.0001
High school	671	12.6	558	13.1	113	10.7	0.65(0.52–0.82)	0.0002
College or higher	188	3.5	179	4.2	9	0.8	0.16(0.08–0.32)	<0.0001
Smoking								
Never smoked	4013	75.6	3320	78.2	693	65.4	reference	<0.001
Ever smoked	1292	24.4	925	21.8	367	34.6	1.83(1.59–2.10)	
Alcohol drinking								
No	4119	77.6	3377	79.6	742	70.0	reference	<0.001
Yes	1186	22.4	868	20.4	318	30.0	1.67(1.43–1.94)	
BMI (Kg/m^2^)								
<18.5	330	6.2	289	6.8	41	3.9	0.65(0.46–0.92)	0.0139
≥18.5 and <23	2184	41.2	1792	42.2	392	37.0	reference	
≥23 and <27.5	2222	41.9	1714	40.4	508	47.9	1.36(1.17–1.57)	<.0001
≥27.5	569	10.7	450	10.6	119	11.2	1.21(0.96–1.52)	0.1055
Family history of TB								
No	5232	98.6	4192	98.8	1040	98.1	reference	0.1126
Yes	73	1.4	53	1.2	20	1.9	1.52(0.91–2.56)	
Close contact with TB patients								
No	5270	99.3	4223	99.5	1047	98.8	reference	0.0135
Yes	35	0.7	22	0.5	13	1.2	2.38(1.20–4.75)	
BCG Scar								
No	3410	64.3	2567	60.5	843	79.5	reference	<.0001
Yes	1895	35.7	1678	39.5	217	20.5	0.39(0.34–0.46)	

Abbreviations: BCG, bacillus Calmette-Guérin; BMI, body mass index; CI, confidence interval; QFT, QuantiFERON-TB Gold In-TubeQFT; OR, odds ratio; TB, tuberculosis.

**Table 2:** The LTBI rate differences among male and female subjects among different age groups.

Age (years)	Female				Male					P
	QFT negative (n)	(%)	QFT positive (n)	(%)	QFT negative (n)	(%)	QFT positive (n)	(%)		
5–9	41	97.62	1	2.38	55	98.21	1	1.79	0.75(0.05–12.27)	*1.00
10–19	131	99.24	1	0.76	160	98.16	3	1.84	2.46 (0.25–23.89)	*0.6308
20–29	195	94.66	11	5.34	179	93.72	12	6.28	1.19(0.51–2.76)	0.6878
30–39	233	88.59	30	11.41	174	87.00	26	13.00	1.16(0.66–2.03)	0.6025
40–49	624	85.13	109	14.87	449	80.18	111	19.82	1.42(1.06–1.89)	0.0189
50–59	466	78.45	128	21.55	374	69.00	168	31.00	1.64(1.25–2.14)	0.0003
60–69	408	78.46	112	21.54	343	67.25	167	32.75	1.77(1.34–2.34)	<.0001
≥70	262	75.29	86	24.71	151	61.63	94	38.37	1.90(1.33–2.70)	0.0004

Abbreviations: CI, confidence interval; OR, odds ratio; LTBI, latent tuberculosis infection; OR, odds ratio; QFT, QuantiFERON-TB Gold In-TubeQFT;

*Fisher exact test;

**Table 3:** Multivariate logistic regression on risk factors for Mycobacterium tuberculosis infection.*

Variables	β	Wald Chi-square	P	OR	95%CI
Intercept	-2.9102	130.0982	<.0001		
Age (every 10 years)	0.0308	122.7828	<.0001	1.03	1.03–1.04
Gender (male)	0.2799	9.4559	0.0021	1.32	1.11–1.58
BMI (≥23 and <27.5 Kg/m^2^)	0.2186	9.441	0.0021	1.24	1.08–1.43
Smoking (Yes)	0.343	12.3513	0.0004	1.41	1.16–1.71
Close contact with TB patients (Yes)	0.3715	4.1486	0.0417	2.10	1.03–4.30
BCG Scar (Yes)	-0.2311	5.3504	0.0207	0.79	0.65–0.97

Abbreviations: BCG, bacillus Calmette-Guérin; BMI, body mass index; CI, confidence interval; OR, odds ratio.

*Age: every 10 years; Gender: female as reference; BMI: BMI ≥18.5 Kg/m^2^ and BMI <23 Kg/m^2^ as reference; None smoking status as reference; None closing contact with tuberculosis patients as reference; No BCG Scar as reference.

## Discussion

Although simple to perform and inexpensive, the tuberculin skin test (TST) need a return of visit and could not distinguish *M*. tuberculosis infection from prior BCG vaccination [13]. However, with higher sensitivity and specificity than TST, interferon-gamma release assays were recommended for the diagnosis of LTBI [12, 14], and interferon-gamma release assays have been adopted for confirmation of the status of LTBI in several studies [15, 16]. In our study, the interferon-gamma release assay by QFT was used to test the status of LTBI.

In this cross-sectional study, we evaluated the rate of LTBI in the rural area of eastern China, and revealed associated factors that would be involved in the risk of LTBI. 5305 subjects provided valid results and showed a LTBI of 19.98% in the rural population. Meanwhile, age, male gender, smoking, overweight and close contacting with tuberculosis were risk factors for LTBI, while BCG vaccination was a protective factor for LTBI.

In our study, we found that subjects with BCG scar were protected from LTBI. BCG vaccination was included in the national immunization system of China since 1978, which means those subjects aged 35 years old or younger (calculated by the time of our investigation) would be protected from LTBI if people vaccinated with BCG. Meanwhile, our data showed that since the age categories above from 30–39 years level, the risks of LTBI were in significance when compared with the age category of 5–9 years. This may implicate that those population with age less than 30 years old might be protected by BCG vaccination from LTBI infection. Review of randomized controlled trials showed that BCG was in effective of protection form LTBI until 10 years [17]. Recently, Roy et al. conducted a meta-analysis of 14 studies showed that BCG was protecting children less than 16 years from LTBI and also from tuberculosis infection to tuberculosis disease [18]. Our study supported the findings and provided more evidence that people aged less than 30 years might be also protected from LTBI and with only a slightly increased rate of LTBI among those aged 20–29 years (5.79%).

Although the increasing trend of LTBI rate was overwhelming along with age, the gender difference of LTBI may further implicate clues for LTBI control in target population. It is reported that the ratio of male to female among tuberculosis patients was 2 to 1 [19], which suggested paying more efforts on tuberculosis control on male subjects.

In our study, we found that LTBI rate among male subjects was significantly higher than that among female subject since the age group of 40–49 year. Ting et al. discussed the gender disparity on the risk of LTBI, and they reiterated that male gender was not contributing to the increased risk of LTBI after controlling confounders of age, smoking status and other clinical factors [20]. However, in the multivariate analysis of LTBI risk factors, we found that male gender still increased the risk of LTBI, which need to be paid more attention for latent tuberculosis control. Except the different view of gender on LTBI risk, smoking habit was an independent risk factor for LTBI among Ting’s study and in other previous association studies [21–23]. Our results also supported the findings. Meanwhile, the passive smoking for children also increased the risk of LTBI [24, 25]. Cigarette smoke exposure inhibited the lung T-cell production of IFN-gamma during stimulation in vitro with anti-CD3 and thus increased the susceptibility to *M*. tuberculosis [26]. Meanwhile, the possible mechanism of smoking inducing increased susceptibility to LTBI was also supported by the evidences found in vitro that smoking impairs macrophage control of *M*. tuberculosis and the nicotine and acrolein were implicated in smoking induced immunosuppression [27]. Thus, control of cigarette smoking would be benefit to tuberculosis control in settings with high LTBI rate.

According to the classification of BMI for Asian people [11], overweight (BMI between 23 and 27.5 Kg/m^2^) was found to elevate the risk of LTBI for the general population in the rural area in our study. Many studies have proved that people with lower BMI was at increased risk of developing tuberculosis disease [28]. However, how BMI affected the *M*. tuberculosis infection was not well described. Studies focused on another particular population, the health care workers, and showed discrepant results of BMI on LTBI risk [29, 30]. Our cross-sectional study with a large sample size provided implications that overweight might be contributed in LTBI increased risk.

Besides the important findings of LTBI in the eastern part of China, several limitations of our study need to be addressed. Firstly, the study population can’t represent the urban population because of the sampling was only conducted in the rural area. Secondly, the QFT method was an indirect method for LTBI diagnosis, and it may not totally represent the existence of *M*. tuberculosis in vivo, because we don’t know how long the immunology of human body to *M*. tuberculosis will last. Meanwhile, the sensitivity of QTF will be decreased for those immunosuppression patients or those people receiving immunosuppressive agent [1]. However, the differentiation ability of the QTF is more convincing in detection *M*. tuberculosis induced infection rather than other *Mycobacteria* when compared with TST method.

Risk factors for developing tuberculosis have been extensively studied previously. However, the risk factors for LTBI were rarely reported, and the importance of revealing the risk factors for LTBI in high LTBI regions was critical for tuberculosis control. We conducted this cross-sectional study with a large sample size to disclose the current LTBI situation in the eastern rural China and the associated risk factors which should be paid more attention under the current tuberculosis control strategy in China, and the similar results could be referred by regions with similar burden of tuberculosis and economic development.

## Supporting Information

S1 Dataset (SAS7BDAT)
